# Renin-angiotensin system inhibitors improve the clinical outcomes of COVID-19 patients with hypertension

**DOI:** 10.1080/22221751.2020.1746200

**Published:** 2020-03-31

**Authors:** Juan Meng, Guohui Xiao, Juanjuan Zhang, Xing He, Min Ou, Jing Bi, Rongqing Yang, Wencheng Di, Zhaoqin Wang, Zigang Li, Hong Gao, Lei Liu, Guoliang Zhang

**Affiliations:** a National Clinical Research Center for Infectious Diseases, Shenzhen Third People's Hospital, Southern University of Science and Technology, Shenzhen, People’s Republic of China; b Shenzhen Bay Laboratory, Shenzhen, People’s Republic of China; c Lead Contact

**Keywords:** COVID-19, hypertension, Renin-angiotensin system, angiotensin-converting enzyme inhibitors, angiotensin II type1 receptor blockers

## Abstract

The dysfunction of the renin-angiotensin system (RAS) has been observed in coronavirus infection disease (COVID-19) patients, but whether RAS inhibitors, such as angiotensin-converting enzyme inhibitors (ACEIs) and angiotensin II type 1 receptor blockers (ARBs), are associated with clinical outcomes remains unknown. COVID-19 patients with hypertension were enrolled to evaluate the effect of RAS inhibitors. We observed that patients receiving ACEI or ARB therapy had a lower rate of severe diseases and a trend toward a lower level of IL-6 in peripheral blood. In addition, ACEI or ARB therapy increased CD3 and CD8 T cell counts in peripheral blood and decreased the peak viral load compared to other antihypertensive drugs. This evidence supports the benefit of using ACEIs or ARBs to potentially contribute to the improvement of clinical outcomes of COVID-19 patients with hypertension.

## Introduction

The COVID-19 outbreak caused by severe acute respiratory syndrome coronavirus 2 (SARS-CoV-2) continues to endanger global health and to hamper the world economy. This outbreak started in December 2019 in Wuhan, Hubei Province. Unfortunately, currently, there is still no specific and effective treatment for COVID-19. Evidence shows that elderly people with SARS-CoV-2 infections and cardiovascular diseases, including hypertension, are at risk of developing severe cases [1]. A hypertension survey from 2012 to 2015 reported that 23.2% of Chinese people ≥18 years of age had hypertension, whereas the prevalence of hypertension was >55% among citizens ≥65 years of age [2]. RAS plays an important role in regulating electrolyte balance and blood pressure and comprises two pathways: the ACE/Ang II/AT1R pathway and the ACE2/Ang (1–7)/Mas receptor pathway [3]. Under normal physiological conditions, the activity of the ACE/Ang II/AT1R axis and the ACE2/Ang (1–7)/Mas receptor axis are in a dynamic equilibrium state, maintaining the normal function of the corresponding system. Similar to SARS, SARS-CoV-2 is believed to invade the host through the cell entry receptor ACE2 [4]. SARS-CoV infections reduce ACE2 expression, resulting in an imbalance between the ACE/Ang II/AT1R axis and the ACE2/Ang (1–7)/Mas receptor axis [5]. Targeting the ACE/Ang II/AT1R axis is a novel therapeutic strategy for hypertension. ACEIs and ARAs not only inhibit the ACE/Ang II/AT1R pathway but also modulate the ACE2/Ang (1–7)/Mas receptor pathway [6]. The dysfunction of the renin-angiotensin system (RAS) has been observed in coronavirus infection disease (COVID-19) patients, but whether RAS inhibitors, such as angiotensin-converting enzyme inhibitors (ACEIs) and angiotensin II type 1 receptor blockers (ARBs), are associated with clinical outcomes remains unknown. Here, we aimed to evaluate the ability of RAS inhibitors to protect against COVID-19 in patients with hypertension.

## Methods

This study was approved by the Shenzhen Third People’s Hospital Ethical Committee. Verbal informed consent was obtained from all patients or patients’ family members. We performed a retrospective review of medical records from hospitalized patients with COVID-19 admitted to the Shenzhen Third People’s Hospital from 11 January to 23 February 2020. The information on patients with hypertension was extracted from all enrolled COVID-19 patients. We reviewed the clinical data extracted from electronic medical records, including clinical symptoms, signs and laboratory findings. A commercial real-time PCR kit (GeneoDX Co., Ltd., Shanghai, China) was used to detect SARS-CoV-2. Samples were considered positive if the cycle threshold value (Ct-value) less than 37 and negative if Ct-value more than 40. Samples with a Ct-value between 37 and 40 require confirmation by retesting. Samples identified as positive by the local laboratory were further validated by the key laboratory of the Shenzhen CDC. The severity of COVID-19 wasidentified during the hospitalization according to the guidelines established by the National Health Commission of the People’s Republic of China. Therapeutic regimens for COVID-19 patients complied with guidelines established by the National Health Commission of the People’s Republic of China. Hypertension was classified as Grade 1, Grade 2 and Grade 3 according to 2018 guidelines of the European Society of Hypertension (ESH). Hypertensive patients with COVID-19 were divided into two subgroups based on antihypertensive drug treatments. Detailed information on the enrolled patients is shown in supplementary Table S1. The SPSS 18.0 software package was used for statistical analysis. Measurement data are expressed as the median and interquartile range (IQR), and the difference between groups was compared by an unpaired t test. Count data are expressed as percentages, and the difference between groups was tested by the Chi-square test. *P *< 0.05 was considered statistically significant.

## Results

A total of 417 COVID-19 patients were admitted to the Shenzhen Third People’s Hospital as of 23 February 2020. Among these patients, 51 (12.23%) had hypertension. Nine patients (17.6%) with Grade 1hypertension did not take any antihypertensive drugs during hospitalization and were excluded from the subsequent analysis. The other 42 patients (82.4%) receiving antihypertensive therapy were included in further studies. The 42 analyzed patients were divided into two groups based on antihypertensive therapies: the ACEI/ARB group (17 patients) included patients treated with ACEI or ARB drugs, and the non-ACEI/ARB group (25 patients) included patients treated with other antihypertensive drugs, including calcium channel blockers (CCBs), β-blockers and diuretics. Eight patients (32%) in the non-ACEI/ARB group and 5 patients (29.41%)in the ACEI/ARB group had other comorbidities, such as type 2 diabetes (T2D) and coronary heart disease (CHD). The vast majority of hypertensive patients had received the current therapeutic regimens shown in supplementary Table S1 for over one year. The blood pressure of patients was well-controlled with the above therapeutic regimens during hospitalization.

The comparison of baseline characteristics between the two groups is summarized in supplementary Table S2. The median age of the analyzed subjects was 64.5 years (IQR, 55.8–69.0 years), and 57.1% of them were males. No significant differences in hypertension grades were observed between the ACEI/ARB group and the non-ACEI/ARB group. Both groups showed similar signs and symptoms. The median number of days from the onset of symptoms to hospital admission was 2.0 days in the non-ACEI/ARB group and 4.0 days in the ACEI/ARB group. Meanwhile, the median number of days from symptom onset to hospital discharge was 16.5 days in the non-ACEI/ARB group and 20.0 days in the ACEI/ARB group. The median heart rate and respiratory rate from the non-ACEI/ARB group were 90.5 bpm and 20.0, respectively. In comparison, the median heart rate and respiratory rate from the non-ACEI/ARB group were 80.0 bmp and 20.0, respectively. All baseline characteristics shown in Table S2 were not significantly different between the two groups.

**Figure 1.**
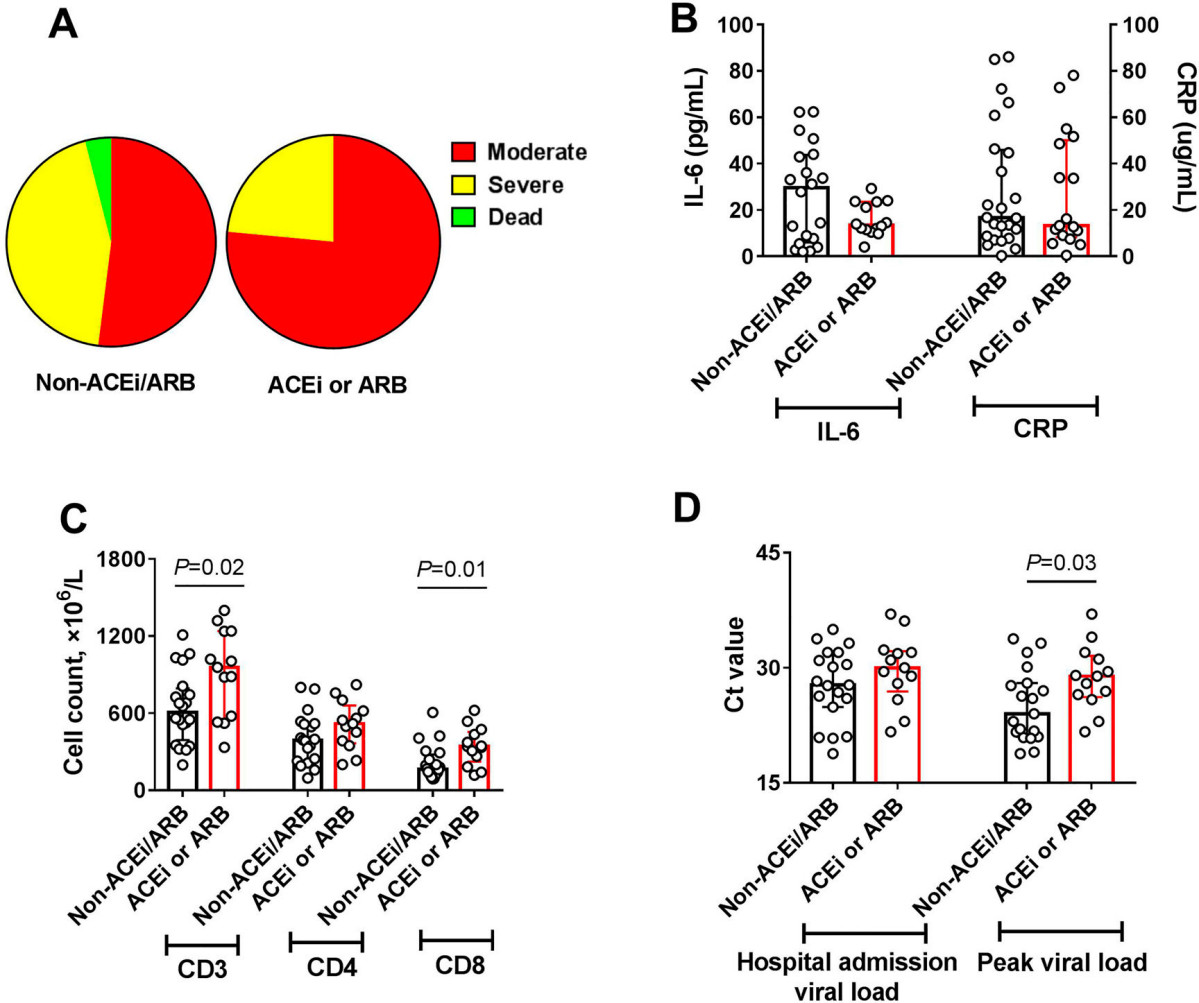
Summarized clinical, inflammatory, immunological, and viral findings in the non-ACEI/ARB group and the ACE/ARB group. (A) The disease severity distribution of the two groups during hospitalization. (B) The levels of IL-6 and CRP in peripheral blood. (C) Absolute numbers of CD3+, CD4+, and CD8+ T cells in peripheral blood. (D) Viral load on hospital admission and maximum value during hospitalization. The data are expressed as the median and IQR. An unpaired t test was used, and *P *< 0.05 was considered significant.

During hospitalization, 12 patients in the non-ACEI/ARB group (48%) were categorized into severe subgroups and one patient died. In contrast, in the ACEI/ARB group, only 4 patients (23.5%) were categorized into severe subgroups and no patients died (Figure 1(A)). The percentage of severe cases in the non-ACEI/ARB group was higher than that in the ACEI/ARB group (12/25, 48.0% vs. 4/17, 23.5%), but this difference was not significant, possibly due to the small number of clinical cases. We next examined the effects of taking ACEI or ARB drugs on laboratory findings of COVID-19 patients with hypertension. As shown in Figure 1(B), there was a trend toward lower IL-6 levels in patients from the ACEI/ARB group. No marked variation in C-reactive protein (CRP) was observed between the two groups (Figure 1(B)). The absolute number of CD3+ and CD8+ T cells in the ACEI/ARB group was significantly higher than that in the non-ACEI/ARB group. There were no significant changes in CD4+ T cell counts between the two groups (Figure 1(C)). In addition, although the viral load was not different between the two groups at hospital admission, the peak viral load during hospitalization in the ACEI/ARB group was significantly lower than that in the non-ACEI/ARB group (Figure 1(D)). Other laboratory findings, such as white blood cell counts, neutrophil counts, platelet counts, and lactate dehydrogenase, are shown in supplementary Table S3, and no significant differences were observed between the two groups.

## Discussion

It was reported that the RAS plays a critical role in regulating hypertension and acute lung injury caused by viruses, such as SARS and H7N9 [5,7]. Changes in RAS activity are related to the pathogenesis of hypertension and inflammatory lung disease. Targeting RAS is an effective antihypertension therapeutic strategy. ACEIs and ARB, which inhibit the ACE/Ang II/AT1R system, are commonly used drugs for hypertensive patients. Recent evidence suggests that hypertensive COVID-19 patients are predisposed to develop severe cases [1]. Thus, it is important to determine the effect of RAS inhibitors on COVID-19 patients with hypertension.

Studies have suggested that COVID-19 patients have increased Angiotensin II compared to healthy people [8]. The abnormal increase in Angiotensin II was related to hypertension and lung failure. In addition, RAS inhibitors have been shown to be associated with reduced mortality in patients with sepsis [9]. Angiotensin II positively regulates the expression of inflammatory cytokines through the activation of AT1R [10]. Excessively high levels of inflammatory cytokines are harmful to the outcomes of COVID-19 patients. Thus, it was suggested that it is beneficial for COVID-19 patients to use ACEIs/ARBs to inhibit the RAS. However, until now, no confirmed clinical evidence has been available. In this study, COVID-19 patients with hypertension were enrolled, and we found that ACEI/ARB therapy attenuated the inflammatory response, potentially through the inhibition of IL-6 levels, which is consistent with the findings that ACEI and ARB therapy alleviated LPS-induced pneumonic injury [11]. For patients with chronic heart failure, it has been proven that ACEI therapy was associated with decreased Th1/Th2 cytokine ratios and inflammatory cytokine production [12]. This study also suggests that ACEI/ARB therapy had a beneficial effect on the immune system by avoiding peripheral T cell depletion. Furthermore, the viral load was reported to be highly correlated with severe lung injury [8]. It was also observed that ACEI/ARB therapy decreased the viral load, but we hypothesize that RAS inhibitors do not directly inhibit viral replication; rather, they play an indirect antiviral role by regulating immune function and inhibiting inflammatory responses, and the mechanism needs to be clarified through in vitro and in vivostudies in the future.

Taken together, this is the first clinical evidence demonstrating that RAS inhibitors improve the clinical outcomes of COVID-19 patients with hypertension, suggesting that these patients could benefit from the persistent or preferential usage of ACEI/ARB for antihypertensive treatment.

## Supplementary Material

Supplemental Material
