# Supplementary material for: Renin-angiotensin system inhibitors improve the clinical outcomes of COVID-19 patients with hypertension
Source: Emerg Microbes Infect. 2020 Mar 31;9(1):757–60. doi: 10.1080/22221751.2020.1746200 (PMC7170368; doi:10.1080/22221751.2020.1746200)
Supplement: Supplemental Material [file TEMI_A_1746200_SM4672.docx]

Table S1 Individual Clinical Information of 42 COVID-19 Patients with Hypertension

| Groups(Non-ACEi/ARB=1; ACEi or ARB=2) | Patients | Sex | Age | Hypertension (Grade) | History of hypertension, y | anti-hypertensive therapy | | | Mean arterial pressure, mm Hg | Comorbidity |
| --- | --- | --- | --- | --- | --- | --- | --- | --- | --- | --- |
|  |  |  |  |  |  | Drugs | Dosage | Duration, m |  |  |
| 1 | Case 1 | M | 66 | 1 | 8 | Amlodipine | 5mg qd | ＞12 | 104.00 | T2DM* |
| 1 | Case 2 | F | 65 | 2 | 5 | L-amlodipine | 2.5mg qd | ＞12 | 94.33 | — |
| 1 | Case 3 | M | 67 | 2 | 12 | Amlodipine  & Metoprolol | 5mg qd  25mg bid | ＞12 | 111.67 | CHD^#^; Hypothyroidism; Gastric cancer |
| 1 | Case 4 | M | 66 | 2 | 30 | Amlodipine  & Metoprolol | 5mg qd  47.5mg qd | ＞12 | 85.33 | CHD |
| 1 | Case 5 | M | 69 | 3 | 10 | Amlodipine  & Bisoprolol | 5mg qd  5mg qd | ＞12 | 115.33 | CHD; Bladder cancer |
| 1 | Case 6 | F | 65 | 3 | 5 | Amlodipine | 5mg qd | ＞12 | 97.00 | — |
| 1 | Case 7 | F | 54 | 1 | 4 | Amlodipine | 5mg qd | ＞12 | 103.00 | — |
| 1 | Case 8 | M | 72 | 3 | 20 | Felodipine | 5mg qd | ＞12 | 98.67 | — |
| 1 | Case 9 | M | 62 | 3 | 10 | Nifedipine | 30mg qd | ＞12 | 117.33 | — |
| 1 | Case 10 | F | 67 | 3 | 15 | Laxidipine  & Metoprolol | 2mg qd  47.5mg qd | ＞12 | 89.00 | CHD |
| 1 | Case 11 | F | 69 | 3 | 10 | Amlodipine | 5mg qd | ＞12 | 132.33 | — |
| 1 | Case 12 | M | 45 | 2 | 8 | Nifedipine | 30mg qd | ＞12 | 123.33 | — |
| 1 | Case 13 | F | 52 | 1 | 5 | Amlodipine | 5mg qd | ＞12 | 103.33 | — |
| 1 | Case 14 | M | 53 | 3 | 13 | Nifedipine | 30mg qd | ＞12 | 134.67 | — |
| 1 | Case 15 | M | 61 | 3 | 10 | Nifedipine | 30mg qd | ＞12 | 115.33 | CHD; T2D |
| 1 | Case 16 | M | 43 | 2 | 9 | L-amlodipine | 2.5mg qd | ＞12 | 107.67 | — |
| 1 | Case 17 | M | 71 | 1 | 10 | Amlodipine | 5mg qd | ＞12 | 97.33 | — |
| 1 | Case 18 | F | 62 | 3 | 30 | Nifedipine  & Metoprolol | 30mg qd  47.5mg qd | ＞12 | 89.00 | CHD; T2D |
| 1 | Case 19 | M | 65 | 3 | 10 | Spironolactone | 20mg qd | ＜1 | 104.00 | T2D |
| 1 | Case 20 | M | 56 | 2 | 5 | Nifedipine | 30mg qd | ＞12 | 123.33 | — |
| 1 | Case 21 | M | 50 | 1 | 1 | L-amlodipine | 2.5mg qd | 1-12 | 113.33 | — |
| 1 | Case 22 | M | 76 | 3 | 16 | Amlodipine | 5mg qd | ＞12 | 114.67 | — |
| 1 | Case 23 | F | 62 | 1 | 7 | Amlodipine | 5mg qd | ＞12 | 109.67 | — |
| 1 | Case 24 | F | 69 | 3 | 30 | Nifedipine | 30mg qd | ＞12 | 100.67 | — |
| 1 | Case 25 | F | 65 | 2 | 5 | L-amlodipine | 2.5mg qd | ＞12 | 115.33 | — |
| 2 | Case 26 | M | 69 | 3 | 12 | Perindopril  & L-amlodipine  & Metoprolol | 2mg qd  2.5mg qd  47.5mg qd | ＞12 | 91.00 | T2D |
| 2 | Case 27 | M | 64 | 3 | 15 | Valsartan and Hydrochlorothiazide tablets | 80/12.5mg qd | ＞12 | 135.67 | CHD; Atrioventricular block |
| 2 | Case 28 | F | 64 | 2 | 8 | Irbesartan  & Metoprolol | 150mg qd  47.5mg qd | ＞12 | 99.33 | — |
| 2 | Case 29 | M | 57 | 3 | 5 | Enalapri &Amlodipine | 10mg qd 5mg qd | ＞12 | 87.00 | — |
| 2 | Case 30 | M | 74 | 3 | 7 | Valsartan  &Nifedipine  & Metoprolol | 80mg qd  30mg qd  47.5mg qd | ＜1 | 116.67 | CHD |
| 2 | Case 31 | M | 49 | 3 | 5 | Valsartan | 80mg qd | ＞12 | 127.00 | — |
| 2 | Case 32 | F | 75 | 3 | 10 | Losartan | 50mg qd | ＜1 | 94.33 | T2D |
| 2 | Case 33 | F | 64 | 2 | 10 | Irbesartan | 150mg qd | ＞12 | 109.67 | — |
| 2 | Case 34 | F | 67 | 2 | 25 | Irbesartan | 150mg qd | ＞12 | 108.67 | — |
| 2 | Case 35 | M | 51 | 3 | 5 | Irbesartan and Hydrochlorothiazide tablets  &Amlodipine | 150/12.5 mg qd  5mg qd | ＞12 | 119.67 | — |
| 2 | Case 36 | F | 55 | 3 | 20 | Valsartan and Amlodipine Tablets | 80/5mg qd | ＞12 | 81.33 | Sjogren's syndrome |
| 2 | Case 37 | F | 57 | 1 | 5 | Olmesartan | 20mg qd | ＞12 | 109.67 | — |
| 2 | Case 38 | F | 69 | 2 | 16 | Valsartan | 80mg qd | ＞12 | 95.67 | — |
| 2 | Case 39 | F | 61 | 2 | 5 | Irbesartan | 150mg qd | ＞12 | 100.00 | — |
| 2 | Case 40 | M | 56 | 1 | 6 | Telmisartan  & Metoprolol | 80mg qd  47.5mg qd | ＞12 | 110.33 | — |
| 2 | Case 41 | F | 79 | 3 | 20 | Irbesartan and Hydrochlorothiazide tablets | 150/12.5 mg qd | ＞12 | 100.33 | — |
| 2 | Case 42 | M | 42 | 3 | 13 | Valsartan  & Metoprolol | 80mg qd  47.5mg qd | ＞12 | 126.00 | — |

Note: *T2DM=Type 2 diabetes mellitus; ^#^CHD= coronary heart disease

Table S2 Baseline Characteristics of analyzed patients in this study

| Clinical characteristics | | No. (%) | | |  |
| --- | --- | --- | --- | --- | --- |
|  |  | All patients (n=42) | Non-ACEi/ARB (n=25) | ACEi or ARB (n=17) | *P* value |
| Age, Median (IQR), y |  | 64.50（55.80-69.00） | 65.00（55.00-68.00） | 64.00（55.50-69.00） | 0.91 |
| Sex |  |  |  |  |  |
|  | M | 24（57.1） | 15（60.0） | 9（52.9） | 0.90 |
|  | F | 18（42.9） | 10（40.0） | 8（47.1） |  |
| Hypertension |  |  |  |  |  |
|  | Grade1 | 8（19.0） | 6（24.0） | 2（11.8） | 0.56 |
|  | Grade 2 | 12（28.6） | 7（28.0） | 5（29.4） | 0.80 |
|  | Grade 3 | 22（52.4） | 12（48.0） | 10（58.8） | 0.71 |
| Signs and symptoms |  |  |  |  |  |
|  | Fever | 31（73.8） | 20（80.0） | 11（64.7） | 0.45 |
|  | Fatigue | 5（11.9） | 1（4.0） | 4（23.5） | 0.15 |
|  | Dry cough | 20（47.6） | 11（44.0） | 9（52.9） | 0.80 |
|  | Expectorant | 11（26.2） | 5（20.0） | 6（35.3） | 0.45 |
|  | Anorexia | 1（2.4） | 0（0） | 1（5.9） | 0.84 |
|  | Myalgia | 6（14.3） | 3（12.0） | 3（17.6） | 0.95 |
|  | Dyspnea | 0（0） | 0（0） | 0（0） | - |
|  | Pharyngalgia | 6（14.3） | 3（12.0） | 3（17.6） | 0.95 |
|  | Bellyache | 3（7.1） | 3（12.0） | 0（0） | 0.38 |
|  | Diarrhea | 3（7.1） | 3（12.0） | 0（0） | 0.38 |
|  | Nausea | 2（4.8） | 1（4.0） | 1（5.9） | 0.65 |
|  | Headache | 1（2.4） | 1（4.0） | 0（0） | 0.84 |
|  | Vomiting | 0（0） | 0（0） | 0（0） | - |
| Onset of symptom to, Median (IQR), d |  |  |  |  |  |
|  | Hospital admission | 3.0（1.0-6.0） | 2.0（1.0-5.5） | 4.0（2.0-7.0） | 0.19 |
|  | Discharged | 17.5（15.5-21.0） | 16.5（13.8-19.5） | 20.0（16.3-21.5） | 0.82 |
| Heart rate, Median (IQR), bpm |  | 86.0（79.0-97.5） | 90.5（85.3-98.0） | 80.0（77.5-93.0） | 0.07 |
| Respiratory rate, Median (IQR) |  | 20.0（19.0-20.5） | 20.0（19.0-21.0） | 20.0（19.5-20.0） | 0.31 |

Table S3 Laboratory Findings of Analyzed Patients in this study on Admission to Hospital

| Laboratory findings | All patients | | Non-ACEi/ARB | | ACEi or ARB | |  |
| --- | --- | --- | --- | --- | --- | --- | --- |
|  | Median (IQR) | No. | Median (IQR) | No. | Median (IQR) | No. | *P* value |
| White blood cell count, ×10^9^/L | 4.83(3.79-6.35) | 42 | 4.87(3.74-6.42) | 25 | 4.68(3.79-6.14) | 17 | 0.82 |
| Neutrophil count, ×10^9^/L | 3.11(2.09-4.46) | 42 | 3.14(2.00-4.41) | 25 | 2.75(2.28-4.47) | 17 | 0.90 |
| Lymphocyte count, ×10^9^/L | 1.15(0.95-1.47) | 42 | 1.12(0.94-1.37) | 25 | 1.38(0.97-1.49) | 17 | 0.94 |
| Monocyte count, ×10^9^/L | 0.46(0.31-0.55) | 42 | 0.43(0.32-0.55) | 25 | 0.40(0.29-0.54) | 17 | 0.58 |
| Eosinophil count, ×10^9^/L | 0(0-0.01) | 42 | 0(0-0.03) | 25 | 0(0-0.01) | 17 | 0.77 |
| Basophils count, ×10^9^/L | 0.01(0-0.01) | 42 | 0.01(0-0.02) | 25 | 0.01(0.01-0.01) | 17 | 0.58 |
| Haemoglobin, g/L | 136.00(126.50-143.50) | 42 | 136.00(126.00-142.50) | 25 | 137.00(125.00-145.00) | 17 | 0.94 |
| Platelet count, ×10^9^/L | 163.50(141.50-191.25) | 42 | 185.00(148.00-250.00) | 25 | 154.00(141.00-177.00) | 17 | 0.05 |
| Prothrombin time, s | 12.30 (11.75-12.70) | 39 | 12.20 (11.25-12.50) | 22 | 12.50(12.00-13.00) | 17 | 0.76 |
| Activated partial thromboplastin time, s | 35.60 (33.45-38.75) | 39 | 36.15 (32.93-38.83) | 22 | 34.80(33.70-36.10) | 17 | 0.56 |
| D-dimer, mg/L | 0.50(0.28-0.77) | 40 | 0.45 (0.28-0.73) | 23 | 0.64 (0.30-0.83) | 17 | 0.35 |
| Albumin, g/L | 42.60(40.15-44.13) | 42 | 42.60(40.95-44.20) | 25 | 43.70(38.25-44.15) | 17 | 0.46 |
| Myoglobin, ug/L | 41.39(27.61-82.75) | 41 | 49.57(27.61-93.15) | 25 | 34.90(22.63-85.19) | 16 | 0.31 |
| Troponin, ug/L | 0.01(0.01-0.01) | 41 | 0.01(0-0.01) | 25 | 0.01(0-0.01) | 16 | 0.91 |
| Procalcitonin, ng/mL | 0.05(0.03-0.07) | 41 | 0.05(0.03-0.07) | 25 | 0.04(0.03-0.07) | 16 | 0.99 |
| Creatine kinase, U/L | 52.00 (46.00-118.00) | 21 | 67.00(48.00-118.00) | 13 | 48.450(42.93-100.20) | 8 | 0.51 |
| Creatine kinase–MB, U/L | 0.86 (0.28-1.42) | 41 | 1.01 (0.54-1.61) | 24 | 0.39(0.28-1.06) | 17 | 0.20 |
| Lactate dehydrogenase, U/L | 317.00(199.50-457.50) | 38 | 227.50(190.50-420.50) | 22 | 406.00(208.50-464.25) | 16 | 0.26 |
| Alanine aminotransferase, U/L | 25.90 (19.43-35.75) | 42 | 26.00 (21.00-36.00) | 25 | 25.800(19.00-34.60) | 17 | 0.72 |
| Aspartate aminotransferase, U/L | 33.05(24.28-41.83) | 42 | 32.00 (22.00-42.10) | 25 | 34.700(27.00-41.00) | 17 | 0.86 |
| Blood urea nitrogen, mmol/L | 4.57 (3.64-5.66) | 42 | 4.58 (3.66-5.76) | 25 | 4.55 (3.63-5.58) | 17 | 0.96 |
| Creatinine, μmol/L | 65.50 (52.50-87.75) | 42 | 66.00 (50.00-80.00) | 25 | 64.00(57.70-98.20) | 17 | 0.63 |
| PaO_2_, mmHg | 83.55(72.80-88.78) | 42 | 83.60(73.75-88.30) | 25 | 82.60(71.45-100.20) | 17 | 0.38 |
| PaO_2_:FiO_2_, mmHg | 391.00(337.30-419.80) | 42 | 389.0(326.50-417.50) | 25 | 393.00(343.00-449.50) | 17 | 0.24 |
| PaCO_2_, mmHg | 37.10(34.60-39.50) | 42 | 36.40(33.90-39.10) | 25 | 37.30(34.90-41.50) | 17 | 0.07 |
